# Arsenic trioxide inhibits Hedgehog, Notch and stem cell properties in glioblastoma neurospheres

**DOI:** 10.1186/2051-5960-2-31

**Published:** 2014-03-31

**Authors:** Dacheng Ding, Kah Suan Lim, Charles G Eberhart

**Affiliations:** 1 Department of Neurosurgery, Xiangya Hospital of Central South University, 87 Xiangya Road, Changsha, Hunan 410078, China; 2 Departments of Pathology, Johns Hopkins University School of Medicine, Baltimore, MD 21287, USA; 3 Oncology, Johns Hopkins University School of Medicine, Baltimore, MD 21287, USA; 4 Ophthalmology, Johns Hopkins University School of Medicine, Baltimore, MD 21287, USA

## Abstract

**Background:**

Notch and Hedgehog signaling have been implicated in the pathogenesis and stem-like characteristics of glioblastomas, and inhibitors of the pathways have been suggested as new therapies for these aggressive tumors. It has also been reported that targeting both pathways simultaneously can be advantageous in treating glioblastoma neurospheres, but this is difficult to achieve *in vivo* using multiple agents. Since arsenic trioxide has been shown to inhibit both Notch and Hedgehog in some solid tumors, we examined its effects on these pathways and on stem cell phenotype in glioblastoma.

**Results:**

We found that arsenic trioxide suppresses proliferation and promotes apoptosis in three stem-like glioblastoma neurospheres lines, while inhibiting Notch and Hedgehog target genes. Importantly, arsenic trioxide markedly reduced clonogenic capacity of the tumor neurospheres, and the stem-like CD133-positive fraction was also diminished along with expression of the stem cell markers SOX2 and CD133.

**Conclusions:**

Our results suggest that arsenic trioxide may be effective in targeting stem-like glioblastoma cells in patients by inhibiting Notch and Hedgehog activity.

## Introduction

Glioblastoma (GBM) are the most common primary adult brain malignancy, and despite some advances in therapeutic options survival remains dismal. One reason suggested for the deadliness of this disease is the presence of treatment-resistant stem-like cancer cells [1-6]. While conventional therapies are thought to target much of the tumor, it is believed that stem-like neoplastic cells survive and go on to regenerate the lesion. We therefore need new therapies targeting these cancer stem cells (CSC) in glioma.

Notch and Hedgehog signaling have been implicated in the survival of CSC in GBM by our group and others, and single agent therapies targeting either pathway have yielded promising results [7-17]. However, single therapies often allow resistance to develop in tumor cells, suggesting that several pathways will need to be targeted simultaneously if we are to eradicate GBM in patients. Our group recently identified one mechanism of cellular resistance to Notch pathway inhibition in GBM: direct upregulation of the Hedgehog pathway through a novel cross talk mechanism. This involved constitutive suppression of Hedgehog activity by direct binding of the Notch mediator HES1 to the GLI promoter [18]. While dual agent therapy with separate compounds targeting both Notch and Hedgehog was able to overcome this problematic therapeutic resistance, toxicity and other issues limit the *in vivo* use of the specific agents tested in our prior study. We have therefore now investigated the potential of a single compound, arsenic trioxide (ATO), to target both Notch and Hedgehog signaling in stem-like glioma cells.

Arsenic trioxide was first used in the treatment of acute promyelocytic leukemia (APL) in China, where one of the potential mechanisms of action involved induction of differentiation of leukemic cells [19,20]. ATO has since been FDA approved for treatment of APL patients for which ATRA failed to work [21]. The effects of ATO were subsequently examined in other tumor types, including multiple myeloma, glioma, neuroblastoma, esophageal carcinoma and prostate cancer, and it has been found to be efficacious in many of these as well [22-27].

The mechanism of action of ATO is not entirely clear, but in many tumors it is thought to function via regulation of various developmental pathways important in cancer. In APL for example, ATO inhibits the oncogenic fusion protein promyelocytic leukemia-retinoic acid receptor α (PML-RAR) [28]. In other tumors such as basal cell carcinoma, ATO is believed to exert its effects by inhibiting the Hedgehog signaling pathway [29]. Finally, in one report using glioma cells grown adherently, ATO was shown to target Notch signaling [30].

A number of studies have looked at the anti-growth effects of ATO in gliomas, however all but one were done using adherent glioma lines grown in high serum [27,30-40]. It has been suggested that glioma cells grown under these conditions are poor models in which to address CSC related issues [41]. We therefore used several serum-free glioblastoma neurosphere cultures to examine the effects of ATO on the growth and survival of stem-like tumors cells, as well as its effects on key developmental pathways such as Notch and Hedgehog. We found that ATO inhibits both of these pathways, along with growth, clonogenicity and stem-cell characteristics in the GBM neurospheres.

## Material and methods

### Cell culture condition and drug preparation

HSR-GBM1, 040622 and 040821 neurosphere lines were kind gifts from Dr. Angelo Vescovi. They include two temozolomide resistant neurosphere cultures (HSR-GBM1 and 040622) and one temozolomide sensitive one (040821) [42]. None are mutated at the IDH1 locus, and HSR-GBM1 and 040622 lack alterations in p53, while 040821 cells have a mutation in p53 exon 7 resulting in substitution of serine for proline in amino acid 278. All are completely methylated at the MGMT locus. Prior studies have shown that all three lines are sensitive to pharmacological Notch blockade [7,18,42], while Hedgehog inhibition has been shown to inhibit growth and clonogenicity of HSR-GBM1 and 040622 (HSR-GBM2) cells [8,18]. GBM neurospheres were maintained in Neurocult Complete Medium supplemented with human epidermal growth factor and human fibroblast growth factor (Peprotech, Rocky Hill, NJ). Arsenic Trioxide (ATO) powder (Sigma, St Louis, Mo-Aldrich) was dissolved in 1 mM sodium hydroxide. Cell number and viability were assessed using the hemocytometer and trypan blue.

### Determination of cell growth

We performed MTS assays to determine growth in viable cell mass. Cells were seeded into 96-well plates at a density of 5000 per well in 100 μl of medium. Cells were then treated with concentrations of arsenic trioxide (ATO) ranging from 0 μM to 5 μM and incubated in 5% CO2 at 37°C. For the MTS readings, 20 μl of MTS solution was added to each well at 24, 48 and 72 hours post plating and incubated for 1 hour. After that optical density was measured by spectrophotometer. The experiments were repeated at least three times for each cell line.

### Cell proliferation assay

BrdU assay was performed to determine proliferation. Cells were treated with concentrations of ATO ranging from 0 μM to 5 μM for 72 hours, and then cytospun onto slides. They were then fixed with 4% formaldehyde in PBS for 15 minutes, and permeabilized with 0.1% Triton/PBST for 15 minutes. Cellular proteins were then denatured with 2 N HCL, washed with PBST (PBS Tween-20), blocked with 5%NGS/PBST for 15 minutes and then incubated in primary antibody against BrdU (Sigma, B2531). Anti-BrdU antibody was used per the manufacturer’s instruction at 1:500 dilution. After washing 3 times with PBST, cells were incubated for 45 minutes in the dark with the appropriate cy-3 conjugated secondary antibody. Cells were then counterstained with 4’,6-diamidino-2-phenylindole (DAPI), mounted with Vectashield (Vector Laboratories), and visualized and pictures taken by fluorescence microscopy.

### Western blot analysis

Proteins were extracted from HSR-GBM1, 040622 and 040821 treated with ATO for 72 hours. The cells were lysed, sonicated until clear, and then centrifuged at 4°C and 15000 rpm for 10 min to remove cell debris. Subsequently, protein separation was performed on a 4-12% SDS-polyacrylamide gel by electrophoresis, and then transferred onto PVDF membranes. Membranes were then probed with primary antibodies overnight.

### Cell cycle analyses

Cells were plated in 6-well plates at a density of 1.5 × 10^5^-2.5 × 10^5^ cells per well and treated with ATO ranging from 0 μM to 5 μM for 72 hours. Cells were then fixed with 70% ethanol for at least 12 hours and stained with Guava Cell Cycle Reagent (Millipore, Billerica, MA). Cell cycle analyses were performed using the Guava PCA machine.

### Clonogenic assays

HSR-GBM1, 040622 and 040821 neurospheres were treated with concentrations of ATO ranging between 0 μM and 5 μM for 72 hours. Neurospheres were then mechanically dissociated into single cells. 20000 viable cells were counted and plated in fresh media containing methylcellulose in 6-well plates. No ATO was present during this growth phase of the clonogenic assay. Pictures of colonies were taken after 10–12 days. 2–3 wells were plated per cell line and 3–4 separate fields were taken per well. Measurements were taken of the widest diameter of each colony and its corresponding right angle diameter and the measurements averaged. The number of colonies that have an average diameter above 100 μM were then counted for each field and graphed.

### Flow cytometric analyses

For CD133 flow cytometric analyses, neurospheres were treated with 0 μM-5 μM ATO for 24 hours and then collected, dissociated, and stained for CD133. Flow cytometry was then performed as previously described [43]. In brief, a PE-conjugated CD133 antibody (Miltenyl Biotec, Auburn, CA), was incubated on ice with the dissociated neurosphere cells for 10 minutes, protected from light. Cells were then washed and resuspended in 500 μl of DMEM/F12 without phenol red containing 1% BSA. Cells incubated with isotype control in parallel were used to set the gates. Cells with PE-readings above isotype control were considered CD133-positive.

### Quantitative real-time PCR analyses

HSR-GBM1, 040622 and 040821 were collected after treatment with 0 μM-5 μM ATO for 24 hours. RNA was then extracted, reversed transcribed, and cDNA levels analyzed by quantitative real-time PCR analysis performed in triplicate with SYBR Green reagents (Bio-Rad, Hercules, CA). Standard curves were used to determine expression levels and all values were normalized to beta-actin. Statistical comparisons are between multiple experiments each with triplicate technical replicates. Primer sequences were as follows: HES1 forward 5′-GTC AAG CAC CTC CGGAAC-3′; HES1 reverse 5′-CGT TCA TGC ACT CGC TGA-3′; HEY1 forward 5′-TCT GAG CTG AGA AGG CTG GT-3′; HEY1 reverse 5′-CGA AAT CCC AAACTC CGA TA-3′; PTCH1B forward 5′-GAC GCC GCC TTC GCT CTG-3′; PTCH1B reverse 5′-GCC CAC AAC CAA GAA CTT GCC-3′; N-Myc forward 5′-CGACCACAAGGCCCTCAGTA -3′; N-Myc reverse 5′-CAGCCTTGGTGTTGGAGGAG-3′ [44]; GLI2 forward 5′-AATCGCACCCACTCCAAC-3′; GLI2 reverse 5′-TGGGGTCTGTGTATCTCTTGG-3′; Human actin- β forward – 5′-CCCAGCACAATGAAGATCAA-3′; and human actin-β reverse: 5′-GATCCACACGGAGTACTTG- 3′.

### Statistical analyses

Statistical significance was evaluated using unpaired, two-tailed Student’s t-test. P-values <0.05 were considered statistically significant. Unless otherwise noted, error bars represent standard error of the mean. All statistical tests were performed using the GraphPad Prism 5 software (GraphPad Software, La Jolla, CA).

## Results

### ATO inhibits growth and promotes apoptosis in glioblastoma neurospheres

**Figure 1:**
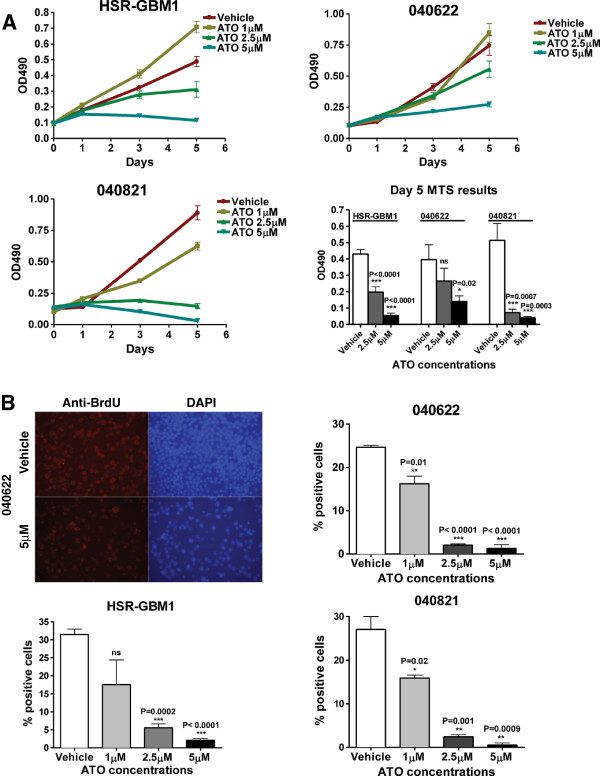
**Arsenic trioxide inhibits glioblastoma neurosphere growth and proliferation. A)** MTS assays showing growth inhibition following ATO treatment in the 040821, HSR-GBM1 and 040622 glioblastoma neurosphere lines, with a bar graph highlighting the significance compared to vehicle. **B)** Representative BRDU staining images showing anti-BRDU staining (red) and DAPI counterstaining (blue) in vehicle and 5 μM ATO-treated 040622 neurosphere cells are shown in the top left panel. The percentage of BRDU positive cells in 040622, 040821 and HSR-GBM1 neurospheres was significantly decreased in all three lines after treatment with either 2.5 μM or 5 μM ATO.

We first examined the effects of ATO on the growth of three glioblastoma neurosphere lines using the MTS assay. In all three lines, we saw a dose dependent reduction in growth over five days (Figure 1a). These studies were performed in technical triplicates at least 4 separate times for each cell line with similar results, and a bar graph containing all data points for the multiple experiments is shown in the lower right panel of Figure 1a. Mean growth inhibition of between 33-86% was seen after treatment at the 2.5 μM ATO levels, and statistically significant 65-92% inhibition of growth was achieved by 5 μM ATO in all three neurosphere lines. To directly determine the effects of ATO on the proliferation of glioblastoma neurospheres, we assessed the number of BRDU positive tumor cells after 72 hours of treatment in the three lines. We identified statistically significant reductions in proliferation of up to 90% using 2.5 uM ATO, and even more pronounced effects with 5 μM ATO (Figure 1B). These data suggest that decreased proliferation accounts for much of the slower growth we observed.

**Figure 2:**
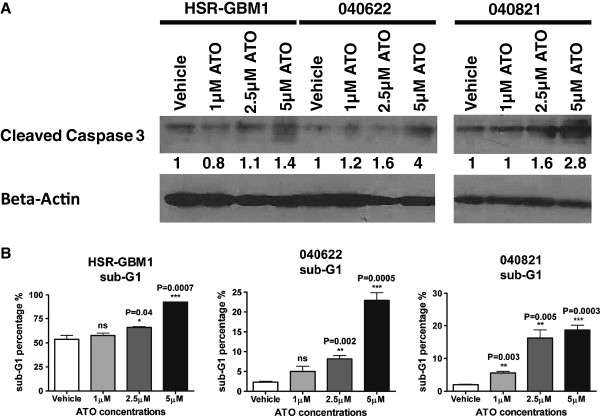
**Arsenic trioxide induces apoptosis in glioblastoma neurosphere cells. A)** Western blots show induction of caspase-3 cleavage in HSR-GBM1, 040622 and 040821 cells following treatment with 2.5 μM or 5 μM ATO. **B)** Cell cycle analysis by flow cytometry performed on neurosphere lines after ATO treatment reveal a significant increase in the sub-G1 population in all 3 neurosphere lines at 2.5 μM and 5 μM ATO. In 040821, there is also a significant increase in the sub-G1 population at 1 μM ATO.

Induction of apoptosis may also mediate some of the anti-tumor effects of ATO. Cleaved caspase 3 levels were increased after ATO treatment in all three lines tested (Figure 2A). In addition, flow cytometric analysis revealed an increase in the sub-G1 fraction of all three lines which was significant at higher levels of the compound (Figure 2B). We also measured GFAP mRNA levels by quantitative RT PCR, but did not detect any increases suggesting increased glial differentiation of tumor cells (data not shown).

### ATO inhibits notch and hedgehog pathway targets

**Figure 3:**
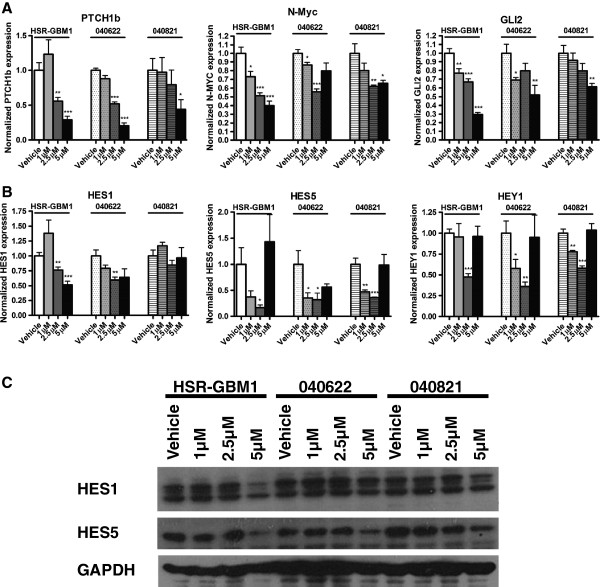
**Arsenic trioxide inhibits Hedgehog and Notch pathway target gene expression. A)** Quantitative PCR analysis revealed that ATO significantly inhibited transcript levels of the Hedgehog pathway target gene PTCH1b at 5 μM in all 3 glioblastoma neurosphere lines compared to vehicle treatment, and in 2 lines after 2.5 μM ATO. ATO also significantly inhibited another Hedgehog pathway target N-Myc in 2 lines at 5 μM, in all 3 glioblastoma neurosphere lines at 2.5 μM, and in 2 lines at 1 μM. GLI2, a 3^rd^ Hedgehog pathway target, was significantly inhibited in all 3 lines at 5 μM ATO, in 1 line at 2.5 μM ATO, and in 2 lines at 1 μM ATO. **B)** ATO also significantly inhibited expression of the Notch target HES1 as compared to vehicle treatment in HSR-GBM1 and 040622 cells, but not in 040821. HES5 and HEY1 transcript levels were also reduced in all 3 glioblastoma neurosphere lines following 2.5 μM ATO treatment, but a non-significant paradoxical increase was seen at the higher 5 μM dose. **C)** ATO inhibited HES1 and HES5 protein expression in all 3 glioblastoma neurosphere lines at 5 μM, compared to vehicle treatment.

Because ATO has been reported to suppress Hedgehog and Notch activity in other tumor types, we examined effects on the two pathways after 24 hour treatment of GBM neurosphere lines. Pathway activity was assessed using quantitative RT PCR to measure levels of the canonical pathway transcriptional targets PTCH1b, GLI2 and N-Myc (Hedgehog) as well as HES1, HES5 and HEY1 (Notch). At least two experimental replicates were performed, each with triplicate technical measurements, and all data points are combined in Figure 3. A statistically significant 40 – 89% inhibition of PTCH1b was noted at the highest ATO dose in all three lines, while the lesser 2.5 μM dose resulted in significant inhibition in two of the three lines (Figure 3A). 2.5 μM ATO also resulted in significant inhibition of another Hedgehog target, N-Myc, in all three GBM lines, while the highest ATO dose resulted in significant inhibition of two of the three lines (Figure 3A). 1 μM ATO resulted in significant inhibition of a third downstream Hedgehog pathway member, GLI2, in HSR-GBM1 and 040622, while 2.5 μM ATO resulted in significant inhibition in GBM1 and 5 μM resulted in significant inhibition in all three GBM lines.

We also observed inhibition of Notch target expression in GBM neurospheres treated with ATO. These changes were not dose dependent in all the lines, but the 2.5 μM level of ATO significantly suppressed HES5 and HEY1 in all three cultures, while the suppression of HES1 was significant in two of three (Figure 3B). Interestingly, the highest levels of ATO (5 μM) resulted in an increase in the mRNA levels of HES5 and HEY1 in several lines. While the cause of this is not clear, we have previously observed paradoxical increases in the mRNA levels of Notch targets when treating brain tumor cells with high levels of gamma-secretase inhibitors that target Notch, and this may represent a feedback or resistance mechanism of some sort in cells surviving maximal therapy (CGE, unpublished data). In addition, ATO also resulted in a dose-dependent decrease of HES1 and HES5 at the protein level, further confirming the effect of ATO on Notch signaling (Figure 3C).

### Clonogenic capacity and stem cell markers are reduced following ATO treatment

**Figure 4:**
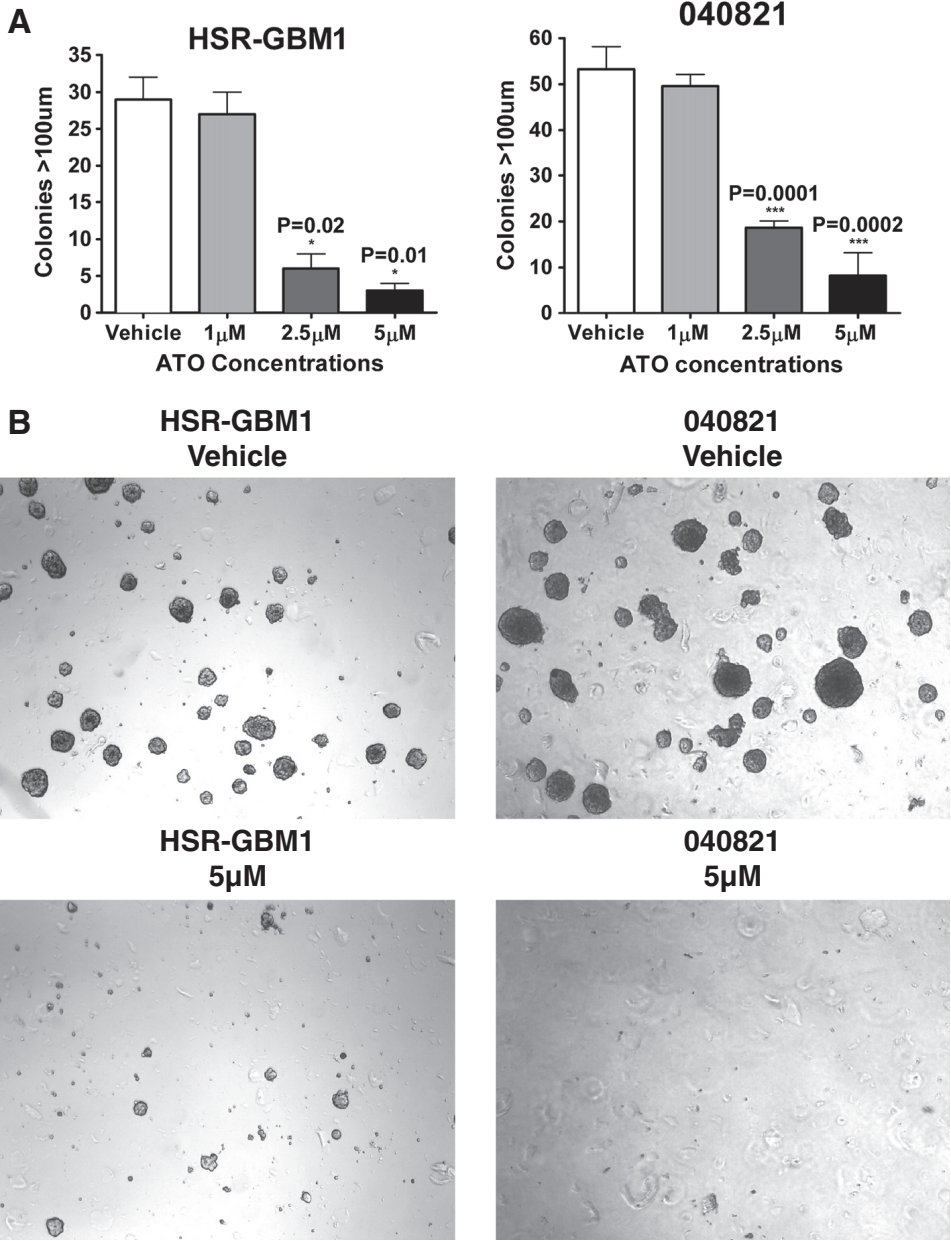
**Arsenic trioxide treatment inhibits clonogenicity in glioblastoma neurosphere cells. A)** Quantification of clonogenicity in equal numbers of single viable HSR-GBM1 and 040821 cells seeded in methylcellulose following 3 days of ATO treatment showed significantly fewer colonies over 100 μM in size. **B)** Representative images of colonies.

We next evaluated the effects of ATO treatment on the clonogenic capacity of glioblastoma cells. Neurosphere lines were treated for three days with ATO, triturated, and counted. Equal numbers of surviving cells were then seeded into methylcellulose and allowed to grow into colonies with no additional treatment. We have previously shown that only large glioblastoma spheres grown in methylcellulose can be serially passaged [45]. The number of spheres over 100 μM in size was significantly reduced following ATO treatment in HSR-GBM1 and 040821 cells (Figure 4), while a modest non-significant reduction was seen in 040622 cells (data not shown). This suggested that the stem-like clonogenic fraction is depleted by ATO treatment.

**Figure 5:**
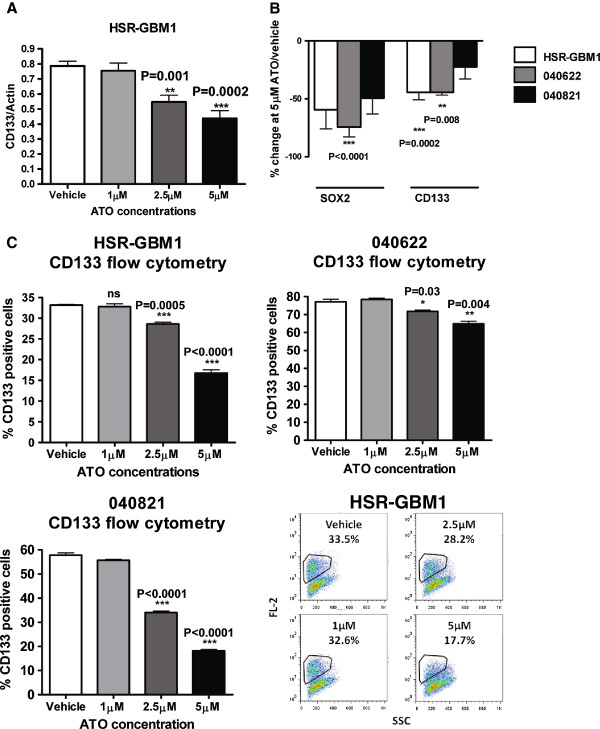
**Arsenic trioxide treatment inhibits the expression of stem-cell markers in glioblastoma neurosphere cells. A)** A representative experiment showing decreased transcript levels of the stem-cell marker CD133 in HSR-GBM1 cells following ATO treatment measured using quantitative real-time PCR. **B)** mRNA expression of the stem-cell markers CD133 and SOX2 was suppressed by ATO. **C)** Flow cytometric analysis of the CD133 positive fraction also revealed variable reductions in this stem-like subpopulation following ATO treatment. Representative flow cytometry plots of CD133-positive cells in vehicle and ATO treated HSR-GBM1 cultures are shown in the lower right panel.

To more directly examine the effects of ATO on stem cell markers, we measured levels of SOX2 and CD133 mRNA after 24 hours of treatment. For the HSR-GBM1 and 040622 lines, a dose-dependent significant inhibition in CD133 mRNA expression was seen (Figure 5A, B). Reductions in SOX2 mRNA levels were also noted in all three lines, and were significant in 040622 (Figure 5B).

We also used flow cytometric analysis to measure the fraction of cells expressing the marker CD133 on their surface. Triplicate analyses showed significant reductions in this stem-like cell fraction at both 2.5 μM and 5 μM levels of ATO in all three lines (Figure 5C). The changes were most pronounced in the HSR-GBM1 and 040821 lines, and relatively modest in the 040622 cells. This experiment was repeated twice with similar results.

## Discussion

Standard GBM treatment includes surgical resection followed by radiotherapy and temozolomide (TMZ) chemotherapy, but rarely results in long term survival [46]. In this study, we focused on the potential of ATO as a therapeutic agent for GBM patients, particularly its ability to inhibit the Notch and Hedgehog pathways which have been shown to play key roles in stem-like glioma cells. While both of these pathways have been shown to be inhibited by ATO in some tumor types [47,48], the ability of this compound to affect them in gliomas is less clear.

Zhen and colleagues treated three adherent GBM lines (U87MG, U251MG and U373MG) with ATO and reported decreased protein levels of the Notch1 receptor and HES1 target, as well as reductions in growth *in vitro* and *in vivo *[30]. They also identified lower levels of the stem/progenitor marker Nestin after treatment, although as noted above high serum lines are not thought to be good models of cancer stem cells in glioma. To our knowledge, no data addressing the effects of ATO on Hedgehog activity in glioma has been published.

We examined the effects of ATO inhibition on Notch and Hedgehog pathways in three GBM neurosphere lines which we have previously shown to contain stem-like fractions that require one or both of these signaling cascades [7,8,18]. We found that ATO inhibits growth and clonogenicity with induction of apoptosis in all three lines. CD133 percentage, as assessed by flow and quantitative real-time PCR, as well as the levels of other stem cell transcripts, were also reduced following ATO treatment. Finally, we found significant reductions in the expression of both Notch and Hedgehog pathway targets.

A recently published study by Wu et al. [47] also examined the effects of ATO on Notch signaling and clonogenic growth in several adherent GBM lines as well as what appeared to be a single neurosphere culture [47]. Like us, they found reductions in stem cell markers and phenotype in the glioma cells following ATO treatment, which they suggest were at least partially due to effects on Notch. They did not examine Hedgehog pathway inhibition. In two studies of ATO treatment in leukemia patients, cerebrospinal fluid levels were 10-17% of those in serum, thus local delivery may be necessary in brain tumor patients [49,50].

In summary, ATO has been previously shown to inhibit the growth of high serum GBM cultures, but little was known about its effects on glioma stem cells or the pathways thought to promote their specification and survival. We found that ATO depletes stem-like cancer cells as defined by surface CD133 expression in all three GBM neurosphere lines tested, with negative effects on overall growth and clonogenicity. We also noted inhibition of both Notch and Hedgehog signaling by ATO, suggesting a potential mechanism for the effects on cancer stem cells and tumor growth. Our findings are consistent with another recent study of ATO and Notch signaling in GBM neurospheres, and together these and prior reports support the development of ATO as a clinical therapeutic agent capable of inhibiting multiple signaling pathways important in stem like glioma cells.

## Competing interests

The authors declare that they have no competing interests.

## Authors’ contributions

DD and KSL performed cell culture and molecular studies, collected and interpreted data, and helped to draft the manuscript. CGE helped with experimental design, interpretation of data and drafted the manuscript. All authors read and approved the final manuscript.
